# Isoflavonoid metabolism in leguminous plants: an update and perspectives

**DOI:** 10.3389/fpls.2024.1368870

**Published:** 2024-02-09

**Authors:** Qilin Yang, Guodong Wang

**Affiliations:** ^1^ Key Laboratory of Seed Innovation, Institute of Genetics and Developmental Biology, The Innovative Academy of Seed Design, Chinese Academy of Sciences, Beijing, China; ^2^ College of Advanced Agricultural Sciences, Chinese Academy of Sciences, Beijing, China

**Keywords:** isoflavonoids, metabolism, human health, nodulation, plant-microbe interactions

## Abstract

Isoflavonoids constitute a well-investigated category of phenylpropanoid-derived specialized metabolites primarily found in leguminous plants. They play a crucial role in legume development and interactions with the environment. Isoflavonoids usually function as phytoalexins, acting against pathogenic microbes in nature. Additionally, they serve as signaling molecules in rhizobial symbiosis. Notably, owing to their molecular structure resembling human estrogen, they are recognized as phytoestrogens, imparting positive effects on human health. This review comprehensively outlines recent advancements in research pertaining to isoflavonoid biosynthesis, transcriptional regulation, transport, and physiological functions, with a particular emphasis on soybean plants. Additionally, we pose several questions to encourage exploration into novel contributors to isoflavonoid metabolism and their potential roles in plant-microbe interactions.

## Introduction

**Figure 1:**
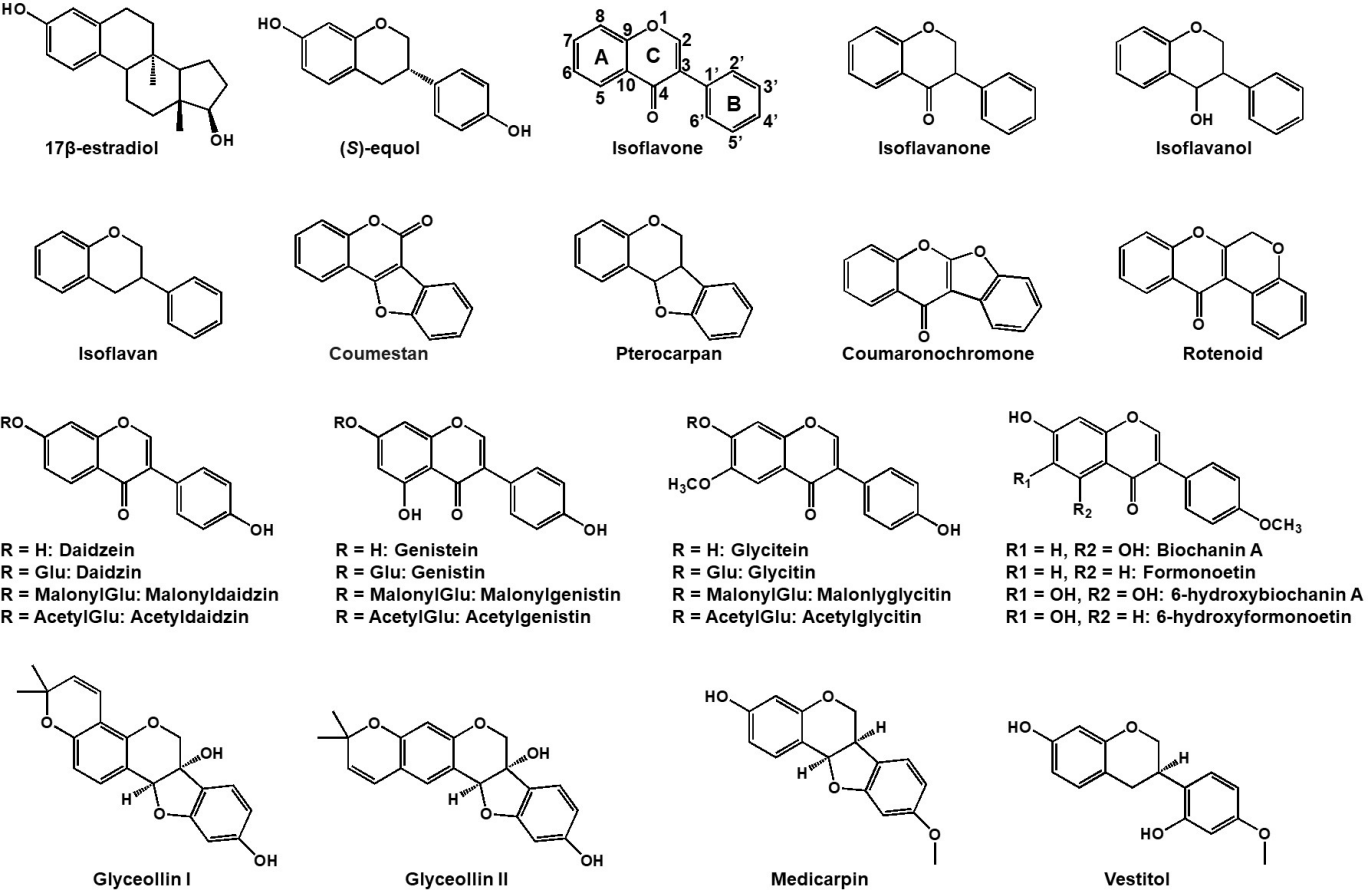
Chemical structures of representative isoflavonoids in leguminous plants. Both 17β-estradiol (estrogen) and daidzein-derived (*S*)-equol have a high affinity for estrogen receptor in human.

Isoflavonoids, primarily found in legumes, are recognized as phytoestrogens owing to their structural and size resemblance to human estrogens (17β-estradiol and daidzein-derived (*S*)-equol, structures see **Figure 1**) (Krizova et al., 2019). Soybeans and soy products are the primary source of these compounds in human diets (Rizzo and Baroni, 2018). Following ingestion, isoflavonoids especially the genistein and daidzein, exhibit the capacity to bind to estrogen receptors, thereby exerting estrogenic or anti-estrogenic effects. (Ariyani et al., 2023). They are thought to confer a protective effect against hormone-related cancers, such as prostate and breast cancer (Boutas et al., 2022; Yu et al., 2023). Besides cancer prevention, isoflavonoids are implicated in averting various diseases, including cardiovascular ailments, Alzheimer’s disease, and possess anti-inflammatory and sterol-lowering properties (Li and Zhang, 2017; Xie et al., 2021; Vina et al., 2022) (**Table 1**). Isoflavonoids extend beyond human health benefits, crucially influencing plant-microbe interactions. They serve as signals in rhizobia-legume symbiosis, triggering nodulation gene expression in rhizobia and enhancing the nodulation process in legumes (Subramanian et al., 2006; Han et al., 2020). Notably, when investigating isoflavonoids in leguminous plants, it is crucial to consider all specialized metabolites as a whole, including other compounds such as saponins (Sugiyama, 2019; Yuan et al., 2023). This review, however, will focus on isoflavonoid distribution, biosynthesis, regulation, transport, and their pivotal roles in plant ecology.

**Table 1:** Pharmacological activities of isoflavonoids from clinical studies.

Isoflavonoid	Dose-time	Pharmacological activities	Ref.
Daidzein	1 tablet/d (6 months)	reducing **lower urinary tract symptoms (LUTS)**	(Tiscione et al., 2017)
Genistein	120 mg/d (12 months)	therapeutics to delay the onset of Alzheimer’s dementia in patients with prodromal **Alzheimer’s disease**	(Vina et al., 2022)
	54 mg/d (24 months)	therapeutics to **glucocorticoid-induced osteoporosis (GIO)**	(Squadrito et al., 2023)
	30 mg/d (3-6 weeks)	influencing the gene expression in **prostate cancer**	(Bilir et al., 2017)
	150 mg/d (1 month)	inhibiting pathways in **human prostate** that drive transformation to a lethal high motility phenotype	(Zhang et al., 2019)
Daidzein/Genistein/Glycitein	136.6 mg/d (5 d/week 2 years)	reducing fibroglandular breast tissue (FGBT), protecting against **breast cancer**	(Lu et al., 2022)
	50 mg/d (8 weeks)	functioning as a complementary treatment for women with migraine to improve **migraine characteristics**	(Babapour et al., 2022)
	52 - 220 mg/d (50 days)	functioning as effective **bone**-preserving agents in **postmenopausal** women	(Pawlowski et al., 2015)
	66 mg/d (6 months)	improving **cardiovascular disease risk (CVR)** markers, and prevent against CVR	(Sathyapalan et al., 2018)

## Chemical structure and distribution of plant isoflavonoids

Isoflavonoids commonly harbor a C6-C3-C6 carbon skeleton—comprising two 6-carbon benzene rings A and C and a 3-carbon heterocyclic ring B (**Figure 1**) (Nabavi et al., 2020). Diverse modifications such as oxidation, glycosylation, acylation, prenylation and others, along with intra-molecular cyclization yield an array of isoflavonoids, including isoflavans, isoflavanones, isoflavanols, coumestans, coumaronochromones, rotenoids, pterocarpans and so on (see **Figure 1**; Veitch, 2007). Thus far, there are more than 2,000 isoflavonoids were isolated and structurally elucidated in plants (Mackova et al., 2006; Veitch, 2007; Al-Maharik, 2019, 2009; 2013). Legume isoflavonoids are comprised of aglycones and glycosides which include glucosides, malonylglucosides, or acetylglucosides. They prevail and translocate within vacuoles of plant cells (Zhao and Dixon, 2010). Different legume plant species may produce different types of isoflavonoids in response to the growing environment. Soybean produces daizein, genistein, glycitein, and derivatives, with glyceollin synthesized in response to pathogens (Ng et al., 2011), while in *Medicago truncatula* and *Lotus japonicus*, the methylated glycones, formononetin and biochanin-A are produced respectively alongside daidzein and genistein. In addition, *M. truncatula* produces medicarpin, while *L. japonicus* yields vestitol through further reduction from medicarpin (Naoumkina et al., 2007; Masunaka et al., 2011).

While isoflavonoids are prominently found in legumes, they are also reported to be isolated in numerous non-leguminous plant families (Reynaud et al., 2005). Over 200 isoflavonoids have been identified in 50+ non-leguminous plant families, spanning Bryopsida, Pinopsida, Magnoliopsida, and Liliopsida classes (Lapcík, 2007).

## Isoflavonoid metabolism in leguminous plants

**Figure 2:**
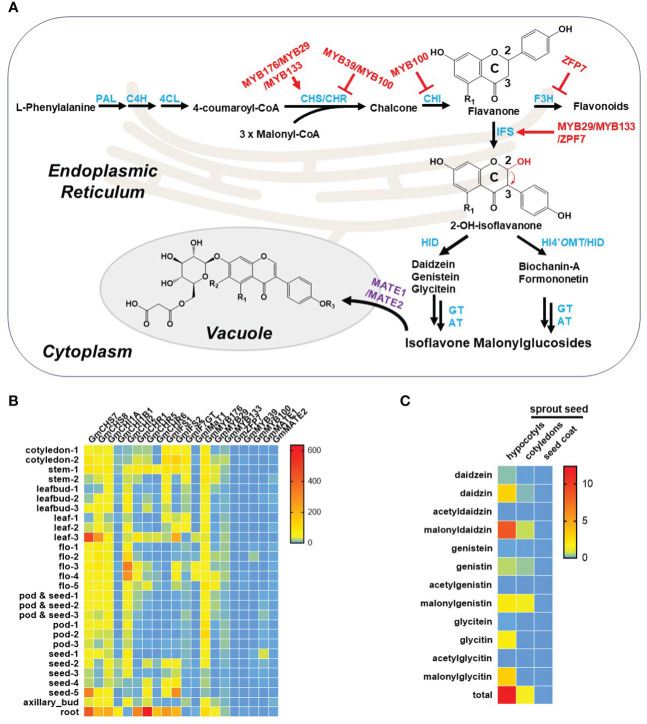
Isoflavonoid metabolism in soybean plants. **(A)** Summary of isoflavonoid metabolism in soybean plant. Notably, R_1_ and R_2_ represent either –H or –OH, and R_3_ represents either –H or –CH_3_ in the chemical structures here. The biosynthetic enzymes, transporters, and transcript factors are highlighted in blue, purple, and red, respectively. Abbreviations: PAL, phenylalanine ammonia lyase; C4H, cinnamic acid 4-hydroxylase; 4CL, 4-coumaroyl-CoA ligase; CHS, chalcone synthase; CHR, chalcone reductase; CHI, chalcone isomerase; F3H, flavanone 3-hydroxylase; IFS, isoflavone synthase; HID, 2-hydroxyisoflavanone dehydratase; HI4’*O*MT, 2-hydroxyisoflavanone 4′-*O*-methyltransferase; GT, glucosyltransferase; AT, acyltransferase. **(B)** Tissue specificity of isoflavonoid known genes in soybean plants. Transcriptome data are extracted from SoyOmics database (https://ngdc.cncb.ac.cn/soyomics/index). **(C)** Distribution of isoflavonoids in seeds. Data are extracted from the work by Yuan et al. (Yuan et al., 2009).

The biosynthetic pathway of isoflavonoids in legumes is currently well investigated, comprising three key phases: the phenylpropanoid pathway, biosynthesis of the isoflavonoid aglycone, and the final production of isoflavonoids (Dixon and Pasinetti, 2010; Sohn et al., 2021). Initially, isoflavonoids originate from the phenylpropanoid pathway, where phenylalanine undergoes a sequential three-step catalytic transformation by phenylalanine ammonia lyase (PAL), cinnamic acid 4-hydroxylase (C4H), and 4-coumaroyl-CoA ligase (4CL) to produce the flavonoid precursor, *p*-coumaroyl-CoA. Subsequently, through the catalytic action of chalcone synthase (CHS), *p*-coumaroyl-CoA condenses with three units of malonyl-CoA to yield naringenin chalcone, a crucial intermediate and the first-rate-limiting step in the flavonoid biosynthetic pathway. Simultaneously, isoliquiritigenin is also formed through the concerted activities of CHS and chalcone reductase (CHR). In soybeans, naringenin chalcone and isoliquiritigenin undergo further enzymatic transformations involving chalcone isomerase (CHI), isoflavone synthase (IFS), and 2-hydroxyisoflavanone dehydratase (HID) to generate three primary isoflavonoid aglycones: genistein, daidzein, and glycitein. However, in other leguminous species such as *M. truncatula* and *L. japonicus*, naringenin chalcone and isoliquiritigenin take an alternative route, giving rise to two distinct 4’-methoxyisoflavonoids, biochanin-A and formononetin. This diversion is facilitated by the involvement of a crucial enzyme, 2-hydroxyisoflavanone 4′-*O*-methyltransferase (HI4’*O*MT). Subsequently, these isoflavonoid aglycones serve as substrates for various modifying enzymes, including glycosyltransferases and acyltransferases, leading to the formation of diverse isoflavonoid derivatives (**Figure 2A**). Additionally, further enzymatic reactions contribute to the synthesis of intricate and biologically active isoflavonoids, such as glyceollin, medicarpin, and vestitol. Notably, two tandem P450 enzymes, C4H and IFS, localized to the endoplasmic reticulum (ER), play a pivotal role in anchoring other isoflavonoid enzymes to the ER through protein-protein interactions, thereby establishing an isoflavonoid metabolon on the ER (Dastmalchi et al., 2016).

IFS, a cytochrome P450 enzyme, is a pivotal rate-limiting determinant governing the transition of intermediates from the flavonoid pathway to the isoflavonoid pathway. This transformation involves the migration of the B-ring from the C2 to the C3 position of the C-ring, succeeded by hydroxylation at the C2 position of the C-ring (**Figure 2A**; Dixon and Steele, 1999; Jung et al., 2000). While IFS is prevalent in legumes, it is not exclusive to this plant family, as evidenced by its presence in non-legumes. For example, IFS has been identified in sugarbeet (*Beta vulgaris*) exhibiting substantial similarity to legume IFS (Jung et al., 2000). In addition to sugarbeet, a wheat-specific *IFS* gene, *TaCYP71F53*, has been recently identified in wheat, setting it apart from legume IFSs by utilizing artocarpanone A as a substrate instead of naringenin or liquiritigenin. The wheat IFS (TaCYP71F53) is responsible for aryl migration coupled with C-ring desaturation of artocarpanone A; however, the legume IFSs are usually responsible for aryl migration and 2-hydroxylation of naringenin or liquiritigenin. Phylogenetic analysis further elucidated that legume IFSs belong to the CYP93 family, while the wheat-specific IFS belongs to the CYP71 family (Polturak et al., 2023). This discovery underscores a parallel gain in the capacity for isoflavonoid production in both soybeans and wheat. In essence, the broad distribution of IFS across diverse plant species highlights its evolutionary significance and the adaptability of distinct plants to engage in the synthesis of bioactive isoflavonoids.

Isoflavonoids synthesized in the ER and cytoplasm often undergo subsequent transport processes, with two main possibilities identified: membrane vesicle-mediated transport and membrane transporter-mediated transport (Zhao and Dixon, 2010). Among membrane transporters, multidrug and toxic compound extrusion (MATE) transporters are considered crucial, particularly in the translocation of isoflavonoids from the cytoplasm to the vacuole. Notably, *GmMATE1* and *GmMATE2*, identified as soy isoflavonoid transporters, facilitate the accumulation of isoflavonoids in soybean seeds. However, studies using the yeast system have revealed that these transporters specifically facilitate the transport of aglycones and glycosides but not malonylglycosides (Ng et al., 2021). Similarly, GmMATE4, localized in vacuole-like membranes, plays a role in mediating the transport of isoflavonoids, including daidzein, genistein, glycitein, and glycitin, into the vacuole for storage. The uptake and efflux of isoflavonoids across the tonoplast or plasma membrane involve various transporters. It is suggested that an unknown ATP-binding cassette (ABC) transporter may be involved in the secretion of soy isoflavonoid aglycones. This inference is supported by the ATP-dependent transport of genistein with and without typical ABC transporter inhibitors (Sugiyama et al., 2007). The intricate interplay of these transport mechanisms adds another layer of complexity to the regulation of isoflavonoid compartmentalization and function within plant cells.

Although the isoflavonoid biosynthesis pathway has been well-investigated in plants, little research focuses on the catabolism of isoflavonoids. Recently, Aoki et al. discovered an isoflavone oxidative catabolic gene cluster in legume root bacteria (*Variovorax* sp.), which belongs to *Comamonadaceae*. The identified catabolism (*ifc*) gene cluster includes at least four functional genes, and the final catabolic products of isoflavones eventually enter the tricarboxylic acid cycle in bacteria. The authors further demonstrated that *ifc* genes are frequently found in bacterial strains isolated from legume plants (Aoki et al., 2023). It can be foreseen that more isoflavone degradation gene clusters will be discovered in near future, especially in plant rhizosphere bacteria.

## Isoflavonoid regulation in leguminous plants

Isoflavonoid biosynthetic enzymes and transporters definitely play a crucial role in determining the production of isoflavonoids. However, the regulatory network involves transcription factors which can directly/indirectly control the expression of isoflavonoid biosynthetic genes, and consequently altering isoflavonoid content. Numerous studies have identified MYB transcription factors as pivotal regulators in the isoflavonoid biosynthesis. For instance, the R1 MYB transcription factor *GmMYB176* was reported to bind with the promoter of *CHS8*, activating *CHS8* expression and enhancing isoflavonoid accumulation (Yi et al., 2010). Similarly, positive regulators like *GmMYB29* (R2R3 MYB) and *GmMYB133* (CCA1-like MYB) were found to activate the expression of *CHS8* and *IFS2*, leading to increased isoflavonoid content (Chu et al., 2017; Bian et al., 2018). However, not all MYB transcription factors exhibit positive regulation; some, such as *GmMYB39* and *GmMYB100*, act as repressors by inhibiting (iso)flavonoid biosynthesis through the repression of upstream genes such as *CHS*, *CHR*, and *CHI* (Liu et al., 2013; Yan et al., 2015). Moreover, some MYB transcription factors do not act alone. They can interact with other transcription factors to regulate the isoflavonoid biosynthesis. For example, GmMYB176 can interact with GmbZIP5, and the co-overexpression of these two genes was shown to increase isoflavonoid content (Anguraj Vadivel et al., 2021). Additionally, other transcription factors, such as the C2H2-type zinc finger protein *GmZFP7*, accelerate isoflavonoid synthesis by promoting the expression of *GmIFS2* and inhibiting the expression of *GmF3H1*, thereby increasing the metabolic flux of isoflavonoids and enhancing their levels (Feng et al., 2023). *GmNAC42-1* was identified as an essential positive regulator of glyceollin biosynthesis, directly binding to the promoters of *IFS2* and *G4DT*, promoting the expression of these two genes, and increasing glyceollin content (Jahan et al., 2019).

In addition to transcription factors, microRNAs might also play a role in mediating the isoflavonoid biosynthetic pathway to regulate isoflavonoid production. *Gma-miR26* and *Gma-miRNA28*, along with their corresponding target genes (*Glyma.10G197900* and *Glyma.09G127200*), were found to be directly related to isoflavonoid content (Gupta et al., 2017). Furthermore, *Gma-miR5030* was identified as a mediator of the expression of the target gene *GmMYB176*, affecting isoflavonoid biosynthesis (Gupta et al., 2019). This intricate regulatory network involving transcription factors and microRNAs adds a layer of complexity to the modulation of isoflavonoid biosynthesis in plants. Notably, the role of microRNAs involved in the regulation of (iso)flavonoid in plants need further experimental validation.

The publicly available transcriptome reveals that genes involved in isoflavone metabolism exhibit higher expression levels in roots and mature seeds compared to other organs, as illustrated in **Figure 2B**. This heightened expression corresponds to increased accumulation of isoflavonoids in these specific tissues. Notably, seeds play a crucial role as a significant source of soy isoflavones for human consumption through direct intake or soy products. The distribution of soy isoflavones within the mature soy seed varies significantly among its different parts. The highest concentration of all isoflavones is observed in the hypocotyl, followed by the cotyledons, while the seed coat contains almost negligible amounts of isoflavones. Furthermore, within the hypocotyl, daidzein and its derivatives exhibit higher levels compared to the other two isoflavones. In the cotyledons, there is an enrichment of genistein and its derivatives (Yuan et al., 2009) (**Figure 2C**).

## Physiological functions of isoflavonoid

Isoflavonoids play pivotal roles in plant ecology, particularly in plant-microbe interactions, functioning as phytoalexins to combat various diseases caused by nematode, oomycete, fungi, bacteria and virus (Lin et al., 2022), and fostering root-rhizobia symbiosis to regulate nodulation (Hammerschmidt, 1999). Notably, transgenic rice expressing the *GmIFS1* gene demonstrated enhanced resistance to the rice blast pathogen, indicating that isoflavonoid biosynthesis, particularly genistein, serves as a phytoalexin in transgenic rice (Pokhrel et al., 2021). In soybeans, glyceollin acts as a crucial phytoalexin induced by cell wall glucan elicitors to resist *Phytophthora sojae*. The silencing of upstream synthesis genes (*IFS* and *CHR*) impairs soybean resistance to *P. sojae* (Ayers et al., 1976; Graham et al., 2007). Glyceollins also exhibit anti-bacterial, anti-nematode and anti-fungal activities besides anti-oomycetes (*P. sojae*) (Ng et al., 2011). Also, the soybean plants with 2-fold increased isoflavonoids through simultaneous knockout of three flavanone genes (*GmF3H1*, *GmF3H2* and *GmFNSII-1*) enhanced the leaf resistance to soya bean mosaic virus (Zhang et al., 2020). In *M. truncatula*, medicarpin synergizes with SA to combat powdery mildew *Erysiphe pisi* and *Rhizoctonia solani* (Gupta et al., 2022).

Isoflavonoids also serve as signals to mediate a symbiotic relationship between legumes and nitrogen-fixing bacteria, primarily Rhizobium, converting atmospheric nitrogen into ammonia for direct plant utilization through the process of biological nitrogen fixation (Yang et al., 2022). Subramanian et al. demonstrated the crucial role of isoflavonoids in nodulation formation, revealing that endogenous production of genistein is sufficient to support nodulation in the soybean hairy root system through RNAi-mediated silencing of the *IFS* and *CHR* genes (Subramanian et al., 2006). During nodulation formation, cells transport (iso)flavonoids, mainly aglycones and glycosides, directly into the apoplast (Biala-Leonhard et al., 2021). In the apoplast, isoflavonoid conjugate-hydrolyzing β-glucosidase (ICHG) hydrolyzes isoflavonoid glycosides, forming aglycones (Matsuda et al., 2023). Subsequently, in the soil, legume roots release these (iso)flavonoid aglycones to attract rhizobia to the rhizosphere. Following this, (iso)flavonoids form a complex by binding to the transcriptional activator NodD protein, activating the transcription of rhizobial nod genes (*nodA*, *nodB*, and *nodC*), and inducing rhizobia to synthesize and secrete the Nod factors, like lipo-chitooligosaccharides (Peck et al., 2006; Del Cerro et al., 2019; Rush et al., 2020).

## Perspectives

In light of the evident of the beneficial impact of isoflavonoids on human health, numerous endeavors have been undertaken to produce isoflavonoids in various chassis, like *Saccharomyces cerevisiae*, *Escherichia coli* and plants (Sajid et al., 2021). Recently, Liu et al. engineered the metabolism of *S. cerevisiae* for *de novo* production of isoflavonoid from glucose. The final optimized strain produces up to 85.4 mg L^−1^ of daidzein and 72.8 mg L^−1^ puerarin (Liu et al., 2021). The efficient synthesis of isoflavonoids using synthetic biology strategies is expected to be a prominent research focus in this field.

**Figure 3:**
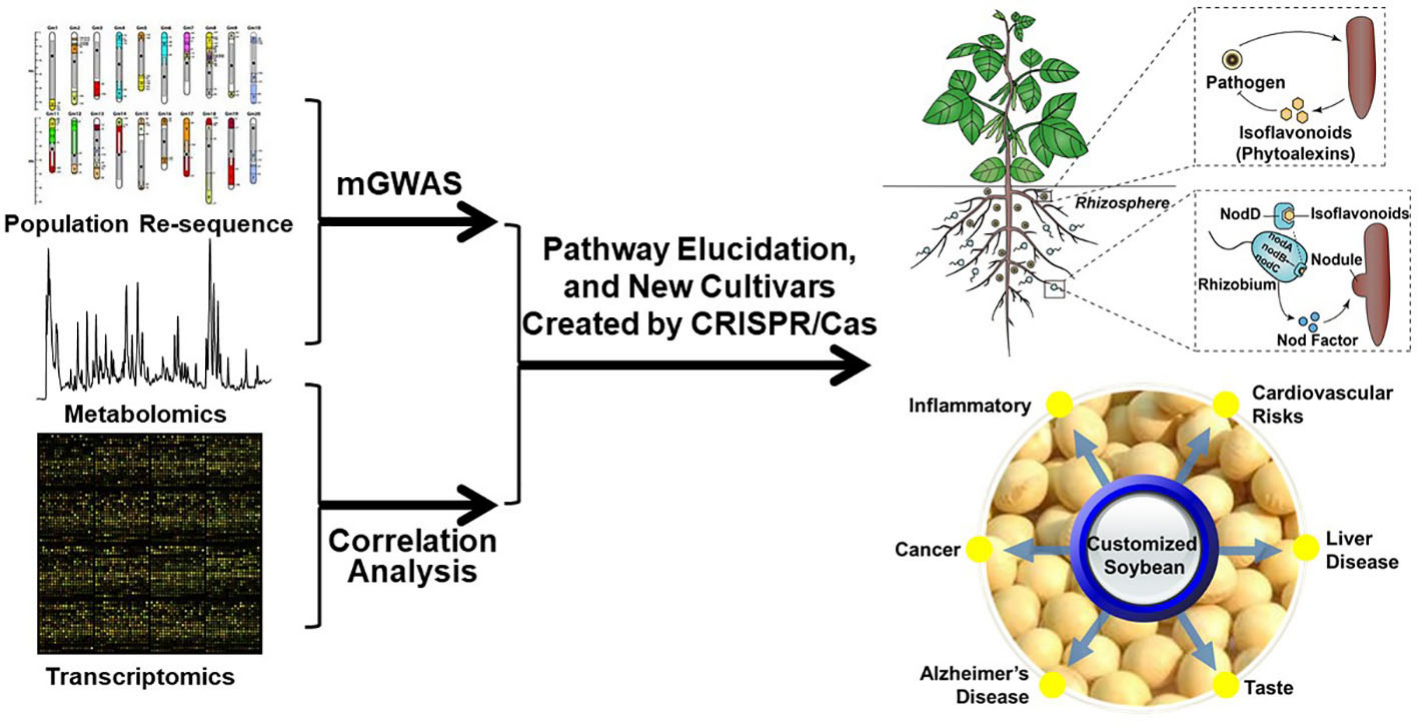
Workflow for isoflavonoid pathway elucidation and their applications in either rhizosphere or seeds.

The exploration of customized soybean seeds with enhanced nutrition and the investigation of the physiological functions of isoflavonoids in plant-microbe interactions offer other exciting avenues for future research (**Figure 3**). Both future researches rely on the comprehensive understanding the biosynthesis and regulation of isoflavonoids and the use of loss-of-function plant materials. Recent years have witnessed the identification of key enzymes in the isoflavonoid biosynthetic pathway, facilitating the utilization of metabolic engineering techniques to expedite the production of higher levels of isoflavonoids for application in the prevention and treatment of associated diseases. Despite the substantial progress, certain isoflavonoid biosynthetic enzymes remain uncharacterized. For instance, although it has been suggested that flavonoid 6-hydroxylase (F6H, a cytochrome P450 monooxygenase belonging to the CYP71D subfamily) may play a role in glycitein synthesis, there is a lack of relevant genetic evidence to substantiate this claim (LatundeDada et al., 2001; Artigot et al., 2013). Continued exploration of these uncharted enzymatic territories holds promise for advancing our understanding and harnessing the full potential of isoflavonoids. Metabolic genome-wide association studies (mGWAS) and quantitative trait locus (QTL), together with co-expression analysis, have emerged as powerful tools in unraveling the complex genetic architecture underlying various biological pathways (Luo, 2015). Given the public availability of soybean-omics databases, these approaches provide a novel and insightful avenue for identifying previously unknown contributors to the isoflavonoid metabolic pathway (Grant et al., 2010; Zheng et al., 2022; Azam et al., 2023; Chu et al., 2023; Liu et al., 2023). The customized soybean seeds thus could be reached by genetic engineering for optimizing isoflavonoid composition and quantity. This process will focus on manipulating key genes involved in the biosynthesis of isoflavonoids and the nutritional knowledge of specific isoflavonoids (**Table 2**). Finally, investing the potential health benefits of customized soybean seeds enriched with specific isoflavonoids could have impact on human health, such as their antioxidant properties, anti-inflammatory effects, and potential role in preventing chronic diseases. For instance, increasing the metabolic flux to daidzein by reducing glycitein branch might be a good target for customized soybean seeds (Zaheer and Humayoun Akhtar, 2017; Alshehri et al., 2021).

**Table 2:** Major QTLs and mGWAS determined for soybean isoflavonoids.

Isoflavonoids	Method	Chromosome	Position	Related Gene	Reference
Total	GWAS	20	43458721^a^	GmMYB29	(Chu et al., 2017)
Glycitein	GWAS	5	36904003	- ^c^	(Chu et al., 2017)
Daidzein Total	GWAS	6	41283321	–	(Chu et al., 2017)
Glycitein	GWAS	11	8262066	–	(Chu et al., 2017)
Daidzein Total	GWAS	8	42737801	GmMPK1	(Wu et al., 2020)
Malonylglycitin	GWAS	11	8147595-8315102	–	(Azam et al., 2023)
Total	GWAS	5	41760764-42234431	–	(Azam et al., 2023)
Genistein Malonylgenitin	GWAS	5	38940662	–	(Kim et al., 2022)
Genistein	GWAS	5	35170270	–	(Kim et al., 2022)
Total	QTL	20	1^b^	GmZFP7	(Feng et al., 2023)
Total	QTL	5	236.4	–	(Cai et al., 2018)
Total	QTL	5	237.1	–	(Cai et al., 2018)
Malonylgenistin	QTL	14	23	GmCHR1	(Pei et al., 2018)
Genistin	QTL	8	0	–	(Pei et al., 2018)
Malonyldaidzin	QTL	19	14	–	(Pei et al., 2018)
Malonylgenistin	QTL	18	158	–	(Pei et al., 2018)
Total	QTL	19	14	–	(Pei et al., 2018)
Malonylglycitin	QTL	11	37	–	(Watanabe et al., 2019)

a unit for mGWAS, base pair (bp).

b unit for QTL, centimorgan (cM).

c -, not determined yet.

As above-mentioned, isoflavonoids play a crucial role as signals mediating plant-microbe symbiosis, particularly in the context of rhizobial interactions. This symbiotic relationship is essential for the ability of legumes to thrive in nitrogen-poor soils, offering a distinct advantage over non-leguminous plants that rely on chemical fertilizers for normal development in low-nitrogen environments. Modifying key enzymes involved in isoflavonoid synthesis in non-leguminous plants holds the potential to promote the establishment of this symbiosis, thereby reducing the dependence on nitrogen fertilizers. The overexpression of *IFS* in transgenic rice has been shown to induce nod gene expression in rhizobia, opening up the possibility of symbiosis (Sreevidya et al., 2006). Furthermore, isoflavonoids can influence the bacterial community composition in the soil. For instance, the bacterial communities in daidzein-treated soils more closely resemble those in the soybean rhizosphere (Okutani et al., 2020). The addition of daidzein enriches the abundance of bacteria in *Comamonadaceae*, a predominant bacterial family in soybean roots. This suggests that manipulating isoflavonoid and other type of plant specialized metabolites could be a strategic approach to change the soil environment, potentially improving crop yields in non-leguminous plants through methods such as crop rotations in nitrogen-deficient conditions (Chen et al., 2019; Sugiyama, 2019; Wang et al., 2023). Understanding the regulation of isoflavonoids in the context of legume-rhizobial symbiosis allows for the development of crops with improved nitrogen fixation capabilities, which is essential for sustainable agriculture by reducing the need for synthetic fertilizers.

## Author contributions

QY: Writing – original draft, Conceptualization. GW: Conceptualization, Funding acquisition, Writing – review & editing.
